# Supplementary material for: The impact of community based continuous training project on improving couples’ knowledge on birth preparedness and complication readiness in rural setting Tanzania; A controlled quasi-experimental study
Source: PLoS One. 2021 Jan 12;16(1):e0244845. doi: 10.1371/journal.pone.0244845 (PMC7802973; doi:10.1371/journal.pone.0244845)
Supplement: S2 File — (DOC) [file pone.0244845.s002.doc]

**QUESTIONNAIRES**

**QUESTIONNAIRES FOR FEMALE PARTNERS**

***Questionnaire 1: Socio-demographic Information and knowledge about birth preparedness***

**Part A: Socio-demographic Information**

**Please tick (√) the appropriate option**

| 1. Age in years in years_____________ | | 1. Age at marriage in years_________ |
| --- | --- | --- |
| 1. Marital status 2. Married ( ) 3. Cohabited ( ) | | 1. Educational status 2. None ( ) 3. Primary level incomplete ( ) 4. Primary level complete ( ) 5. Secondary or higher ( ) |
| 1. Employment status 2. Employed ( ) 3. Unemployed ( ) | | 1. Religion 2. Christian ( ) 3. Muslims ( ) 4. Others specify………………………… |
| 1. Ethnic group 2. Fipa ( ) 3. Mambwe ( ) 4. Others specify……………………… | 1. Economic status 2. Use less than one dollar per day ( ) 3. Use more than one dollar per day ( ) | |
| 1. Do you own radio? 2. Yes ( ) 3. No ( ) | | 1. Do you own mobile phone? 2. Yes ( ) 3. No ( ) |
| 1. Characteristic of a nearby health facility 2. Dispensary ( ) 3. Health center ( ) 4. Hospital ( ) | | 1. What is the walking distance to a nearby health facility? 2. Less than one kilometer ( ) 3. One kilometer-5kilometers ( ) 4. More than five kilometers ( ) |
| 1. Are you covered with health Insurance (NHIF or CHF)? 2. Yes ( ) 3. No ( ) | | 1. Pregnancy history 2. Primigravida ( ) 3. Para 1-Para 4 ( ) 4. Para 5+ ( ) |
| 1. Age at 1^st^ delivery (years)___________ | | |
| 1. Current pregnancy characteristics 2. Planned ( ) 3. Unplanned ( ) | | 1. Did you have prior pre-term delivery? 2. Yes ( ) 3. No ( ) 4. Not applicable ( ) |
| 1. Did you have a prior C-section? 2. Yes ( ) 3. No ( ) 4. Not applicable ( ) | | |

**Part B: Knowledge on Birth Preparedness**

Now I would like to ask you some questions about pregnancy and childbirth. Specifically, I am going to be asking you questions about three different phases that women go through when having a child. These phases are the period of being pregnant, the period of labor and birth, and the period immediately after the birth of the child (JHPIEGO, 2004).

1. In your opinion, can unforeseen problems related to pregnancy occur during any pregnancy or childbirth that could endanger the life of a woman?
2. Yes ( ) b) No ( )
3. In your opinion, what are some serious health problems that can occur during pregnancy that could endanger the life of a pregnant woman?
4. _____________ d) _________________
5. _____________ e)___________________
6. ______________ f) __________________
7. In your opinion, could a woman die from [this problem] any of these problems?
8. Yes ( ) b) No ( )
9. In your opinion, what are some serious health problems that can occur during labor and childbirth that could endanger the life of a pregnant woman?
10. _____________ d) _________________
11. _____________ e)___________________
12. ______________ f) __________________
13. In your opinion, could a woman die from [this problem] any of these problems?
14. Yes ( ) b) No ( )
15. In your opinion, what are some serious health problems that can occur during the first 2 days after birth that could endanger the life of the woman?
16. _____________ d) _________________
17. _____________ e)___________________
18. ______________ f) __________________
19. In your opinion, could a woman die from [this problem] any of these problems?
20. Yes ( ) b) No ( )
21. Now, I would like to ask you a few questions about the health of newborn babies. In your opinion, what are some serious health problems that can occur during the first 7 days after birth that could endanger the life of a newborn baby?
22. _____________ d) _________________
23. _____________ e)___________________
24. ______________ f) __________________
25. In your opinion, could a newborn baby die from [this problem] any of these problems?
26. Yes ( ) b) No ( )
27. Have you ever heard the term “birth preparedness”?
28. Yes ( ) proceed to question 29 b) No ( ) proceed to question 30
29. Where did you hear about birth preparedness?
30. From health worker ( )
31. From the media ( )
32. From a family member ( )
33. Others specify_________________________________________
34. In your opinion, what are some things can be prepared for birth?
35. _____________ d) _________________
36. _____________ e)___________________
37. ______________ f) __________________
38. When a pregnant woman has to start antenatal clinic?
39. Below 16 weeks gestation age ( )
40. 17-24 weeks ( )
41. Above 24 weeks ( )
42. What are the recommended antenatal visits
43. Four or more ( )
44. Three ( )
45. Two ( )
46. One ( )
