# Supplementary material for: The impact of community based continuous training project on improving couples’ knowledge on birth preparedness and complication readiness in rural setting Tanzania; A controlled quasi-experimental study
Source: PLoS One. 2021 Jan 12;16(1):e0244845. doi: 10.1371/journal.pone.0244845 (PMC7802973; doi:10.1371/journal.pone.0244845)
Supplement: S3 File — (DOC) [file pone.0244845.s003.doc]

**MADODOSO**

**Dodoso 1: Taarifa za msingi na uelewa juu ya maandalizi ya kujifungua**

Sehemu ya A: Taarifa za msingi

**Weka alama (√) panapohusika**

1. Umri wako kwa miaka ---------
2. Umri wa kuoa/kuolewa kwa miaka--------------------
3. Hali ya ndoa
4. Nimeolewa ( )
5. Ninaishi na mwanamme/mwanamke ( )
6. Kiwango cha elimu
7. Sijapata elimu rasmi ( )
8. Nimeudhuria baadhi ya madarasa ya shule ya msingi ( )
9. Nimemaliza elimu ya msingi ( )
10. Nimemaliza elimu ya sekondari au zaidi ( )
11. Hali ya ajira
12. Nimeajiriwa ( )
13. Sijaajiriwa ( )
14. Dini yako
15. Mkristu ( )
16. Muislam ( )
17. Nyingine taja…………………………
18. Kabila lako
19. Fipa ( )
20. Mambwe ( )

Jingine taja………………………

1. Hali yako ya uchumi
2. Ninatumia chini ya Tshs 2000 kwa siku ( )
3. Ninatumia zaidi ya Tshs 2000 kwa siku ( )
4. Je unamiliki redio?
5. Ndiyo ( )
6. Hapana ( )
7. Je unamiliki simu?
8. Ndiyo ( )
9. Hapana ( )
10. Kitua cha huduma za afya kilicho karibu nanyi ni:-
11. Zahanati ( )
12. Kituo cha afya ( )
13. Hospitali ( )
14. Kuna umbali gani kutoka nyumbani kwenu mpaka kituo cha kutolea huduma za afya kilicho karibu?
15. Chini ya kilometea moja ( )
16. Kilometa moja mpaka tano ( )
17. Zaidi ya kilometa tano ( )
18. Je una bima ya afya?
19. Ndio
20. Hapana
21. Umezaa mara ngapi?
22. Hii ni mimba ya kwanza ( )
23. Nimezaa mara 1—4 ( )
24. Nimezaa mara tano au zaidi ( )
25. Ulizaa mtoto wa kwanza ukiwa na umri gani (miaka)----------------
26. Mimba ya sasa ilipangwa?
27. Ndio ( )
28. Hapana ( )
29. Je umewahi kujifungua kabla ya wakati?
30. Ndio ( )
31. Hapana ( )
32. Sihusiki ( )
33. Je umewahi jifungua kwa njia ya upasuaji?
34. Ndio ( )
35. Hapana ( )
36. Sihusiki ( )

| **B: Uelewa kuhusu maandalizi ya kujifungua**  Sasa nitakuuliza maswali kuhusu ujauzito, kujifungua na baada ya kujifungua | |
| --- | --- |
| 1. Je unafikiri unafikiri matatizo yasiyotegemewa yanayoweza kujitokeza wakati wa mimba, kujifungua au baada ya kujifungua na yanaweza kuhatarisha maisha ya mama na mtoto? 2. Ndiyo ( ) b) Hapama ( ) | |
| 1. Ni dalili zipi za hatari zinazoweza kutokea wakati wa mimba zinazoweza kuhatarisha maisha? | |
| a)_______________________________ | d)__________________________________ |
| b)_______________________________ | e)__________________________________ |
| c)_______________________________ | f)__________________________________ |
| 1. Je unafikiri dalili hizo za hatari (swali la 20) zinaweza kupelekea kifo? 2. Ndiyo ( ) b) Hapana ( ) | |
| 1. Je ni dalili zipi za hatari zinazoweza kutokea wakati wa uchungu na kujifungua zinazoweza huhatarisha maisha ya mama? | |
| 1. _____________________________ | d)____________________________ |
| 1. _____________________________ | e)______________________________ |
| 1. ____________________________ | f)_____________________________ |
| 1. Je dalili hizo (swali la 22) zinazoweza kujitokeza wakati wa uchungu nakujifungua zinaweza kusababisha kifo kwa mama au mtoto? 2. Ndiyo ( ) b) Hapana ( ) | |
| 1. Ni dalili zipi za hatari zinazoweza kutokea kipindi cha siku arobaini na mbili baada ya kujifungua? | |
| 1. _____________________________ | d)____________________________ |
| 1. _____________________________ | e)______________________________ |
| 1. ____________________________ | f)_____________________________ |
| 1. Je dalili hizo za hatari (zilizotajwa swali la 24) zinaweza zikasababisha kifo cha mama? 2. Ndiyo ( ) b) Hapana ( ) | |
| 1. Sasa , Ningependa kukuuliza maswali machache kuhusu afya ya mtoto mchanga.Kwa maoni yako ni dalili zipi za hatari zinazoweza kumtokea mtoto mchanga kwa kipindi cha siku saba baada ya kujifungua? | |
| 1. _____________________________ | d)____________________________ |
| 1. _____________________________ | e)______________________________ |
| 1. ____________________________ | f)_____________________________ |
| 1. Je unafikiri dalili hizo za hatari zinaweza kusababisha kifo cha mtoto mchanga? 2. Ndiyo ( ) b) Hapana ( ) | |
|  | |
|  | |
| 1. Kwa maoni yako unafikiri mpango wa maandalizi ya kujifungua na kukabiliana na dharura unahusisha mambo gani? | |

| a)________________________ | b)______________________________ |
| --- | --- |
| c)______________________ | d)________________________________ |
| e)_______________________ | f)________________________________ |
| 1. Ni wakati gani mama mjamzito anapaswa kuanza kliniki ya mimba? 2. Chini ya wiki ya wiki 16 3. Wiki 17-24 4. Zaidi ya wiki 24 | 1. Mama mjamzito anatakiwa kuhudhuria kliniki ya mimba mara ngapi? 2. Mara nne au zaidi 3. Mara tatu 4. Mara mbili 5. Mara moja |
