# Supplementary material for: The impact of community based continuous training project on improving couples’ knowledge on birth preparedness and complication readiness in rural setting Tanzania; A controlled quasi-experimental study
Source: PLoS One. 2021 Jan 12;16(1):e0244845. doi: 10.1371/journal.pone.0244845 (PMC7802973; doi:10.1371/journal.pone.0244845)
Supplement: Attachment — Submitted filename: Response to reviewers Comments.doc [file pone.0244845.s004.doc]

Point to point response to reviewers’ comments

| Comment | Response |
| --- | --- |
| 1. Please ensure that your manuscript meets PLOS ONE's style requirements, including those for file naming. The PLOS ONE style templates can be found at | The manuscript meets PLOSE ONE style |
| 2. Please include additional information regarding the survey or questionnaire used in the study and ensure that you have provided sufficient details that others could replicate the analyses. For instance, if you developed a questionnaire as part of this study and it is not under a copyright more restrictive than CC-BY, please include a copy, in both the original language and English, as Supporting Information. | The questionnaire was adopted and modified from Jhpiego. Monitoring birth preparedness and complication readiness: tools and indicators for maternal and newborn health. 2004;1–338. Available from: http://pdf.usaid.gov/pdf_docs/PNADA619.pdf  The tool in both language (English and Swahili) is attached as a supporting Information |
| 3. [NO - Include this sentence at the end of your statement: The funders had no role in study design, data collection and analysis, decision to publish, or preparation of the manuscript.].   At this time, please address the following queries:  Please clarify the sources of funding (financial or material support) for your study. List the grants or organizations that supported your study, including funding received from your institution.  State what role the funders took in the study. If the funders had no role in your study, please state: “The funders had no role in study design, data collection and analysis, decision to publish, or preparation of the manuscript.”  If any authors received a salary from any of your funders, please state which authors and which funders.  If you did not receive any funding for this study, please state: “The authors received no specific funding for this work.”  Please include your amended statements within your cover letter; we will change the online submission form on your behalf. | This work is part of my PhD project. The University of Dodoma sponsored the tuition fee and field allowance. Publication fee was not covered in the sponsorship.  The funders had no role in decision to publish, or preparation of the manuscript  The authors received no specific funding for this work |
| 4. Please include a caption for figure 4 (Figure 3 caption included 2x). | The caption for figure 4 is included; the repeated figure three was a typing error. It was meant to be figure 4 |
| 5. We note you have included a table to which you do not refer in the text of your manuscript. Please ensure that you refer to Table 2 in your text; if accepted, production will need this reference to link the reader to the Table. | Table 2 is referred in the text line 260 |
| **Reviewer 1** |  |
| 1. Line 53 and 54: should be clear and specific where these deaths occurred | Authors agree with the comment and correction is made |
| 2. Line 168: correct the name from Sumawanga to Sumbawanga | Authors agree with the correction and it is done |
| 3. Line 171: what was the interval used in the systematic sampling in the selection of the households? | No specific interval was used but rather pre determine direction of selection of household after random selection of the first household. |
| 4. Line 172: state the inclusion criteria for the study population | The inclusion criteria are mentioned |
| 5. Line 173: who was responsible for the assessment of signs and symptoms of the pregnancy in each household visited? This needs to be clarified and the signs and the symptoms assessed as well. | Research assistants who were nurses did the interview and confirmed the test |
| 6. Line 185: check the value of the power at 90% is not 0.84, please confirm and correct. | The sample size calculation is removed as the portion is published |
| 7. Line 203: the couples were only visited for two days irrespective of the gestation age; how will this intervention have impact for a couple visited at early stages of gestation age? | The study reports a part of the study  One of the components of the intervention was followed up. They were followed and assessed for three main behaviors which were birth preparedness, male involvement and use of health facilities for antenatal services, delivery services and postnatal services  In addition they were supplied with pictorial reference materials |
| 8. Line 204: you skipped what happened in step four and went to fifth step, please correct | What happened in step four is included |
| 9. Line 210: was there any consenting process conducted to the participants? This needs to be stated before the start of data collection. Was the data collected using paper questionnaire or electronically? The author should clarify this. Were there also participants below age 18? | Before participation into the study couples were counseled and upon their consent, they were included in the study (Line 244-Line 246)  Upon consent to participate, there record were taken to the nearby health facility where their behavior were recorded as they acted  No electronic data collection was used but an interviewer administered questionnaire |
| 10. the results were well described, how were the assumptions of linear regression verified. the authors also needs to explain whether any statistical method was used to select variables for the multivariable analysis | Variables which showed significant correlation with knowledge about birth preparedness were entered into the regression model |
| 11. Table 1: what was the lower age limit of the participants that were interviewed? | The lowest age was 16 years. |
| 12. Table 1: Please indicate the exactly P-values whether significant of or not instead of leaving them blank and showing significant one with asterisk. Indicate the number of participants in each of the groups, and the n (%) at the top of each column to show what the values in the table mean | The column of p value is removed in this table because the aim of the table is to describe the characteristics of the study respondents.  Authors agree with the observation and n(%) are added in each column |
| 13. Table 2: add the p-values, show the lower limit of the gestation age | The p-values and lower gestation age is added to the table |
| 14. Table 3: add the exact p-values to the table | The exact p-values is added to the table |
| 15. Were there any strength and limitation for this study? This needs to be explained. | Strength and limitation of the study is added in the last two paragraphs after discussion (Line 236- Line 449) |
| **Reviewer 2** |  |
| 1. The authors published a protocol paper with the following citation: "Moshi FV, Kibusi SM, Fabian F. The Effectiveness of Community-Based Continuous Training on Promoting Positive Behaviors towards Birth Preparedness, Male Involvement, and Maternal Services Utilization among Expecting Couples in Rukwa, Tanzania: A Theory of Planned Behavior Quasi-Experimental Study. J Environ Public Health. 2018;2018:1293760. Published 2018 Sep 27. doi:10.1155/2018/1293760" However, the authors do not reference the protocol paper in this manuscript. It is unclear if these are full related studies. If the paper previously published, it would be helpful to reference the methods and then reduce the detail in the introduction and methods sections. For example, the sample size calculation and equations could be removed and referenced. | Yes, the protocol of the study is published. This manuscript is a part of the findings from the protocol. Authors agree with the suggestion and the protocol is cited in the sample size calculation |
| 2. Please clarify how couples were randomized to receive the intervention or not; or were villages randomized to the intervention? It is clear from the description how the districts and villages were selected. | Randomization was not possible in this intervention; there were a lot of limitations one of which was contamination of the intervention. Applying random assignment to neighbors was not possible  It was a quasi experimental study where only the districts were randomly assigned to either intervention or control |
| 3. Please add details in the Methods about the timing of data collection with respect to the pre-test assessment and post-test assessment. These details are included in the discussion (Lines 403-405). Can the authors discuss potential recall bias or influence of the birth experience and outcomes on the post-test results? | A sentence is added in the data collection to explain the timing for data collection for both pre test assessments (Line 223 to Line 225)  Limitation of the study is added after the discussion where recall bias as one of the limitation is discussed (Line 436 to Line 448) |
| 4. Given that the authors interviewed the same couples pre and post intervention, it seems a difference-in-differences analysis would be appropriate. Can the authors discuss why they have not used this approach? Further it would streamline the Tables 3-7. Figures 3 and 4 also present similar information to Tables 3-7; please consider putting one or the other in the supplementary information. | Yes the same couple was assessed pre pre-post in both groups. Difference-in-difference analysis was used.  The result section is organized into three main parts. The first part is the comparison between groups pre- assessment and post assessment. The second part of the results is the comparison within the group, pre- post assessment differenced between both groups. The third part is the analysis of the predictors of difference in mean score. For every respondent the difference score was computed. This score was treated as a new variable. Predictors of change were established using multiple liner regression.  Authors think allowing all tables to be in the study could still hold water but we are looking forward for more opinion |
| 5. In the abstract, please consider adding the baseline knowledge score so readers can understand more clearly the impact of the intervention. | Authors agree with the suggestion |
| 6. In line 96, there is a reference "(martin)" that needs a full citation. Please update. | The reference is inserted |
| 7. In lines 104 to 121, the authors present several programs for improving pregnant women's outcomes. This contrast and comparison presented may be more useful in the Discussion section to set the results from this research in context. | The introduction of several interventions in the background was placed there to introduce the current intervention. If moved can create discontinuity in the background |
| 8. In lines 196-197, please provide clarification as how the doubling of the control arm increases comparability. | Authors aimed at having groups with similar characteristics; avoiding having one wing with older population and other group with younger population |
| 9. Please provide more clarity to the actual intervention that was applied; lines 198-207 are more about the measurement of the intervention, but not about the intervention for the couple. This information would be helpful to the readers. | Actual clarification is added line 218-line 223 |
| 10. Table 4: Please clarify if the mean (SD) knowledge is a score or mean number of danger signs assessed. Further, is there a range associated with each assessment? Further, please limit to 2 significant digits for the mean and SD of scores. | It is a mean knowledge score. One point for each recalled danger sign or what to prepare for birth/emergency  Decimal points are restricted to two places |
| 11. Figures 3 and 4 presentation as line charts make the reader compare scores across domain as opposed to pre/post scores. Consider a bar chart for pre/post scores next to each other. | Authors agree with the suggestion and the figure is changed to chart for pre/post |
