# Supplementary material for: The impact of community based continuous training project on improving couples’ knowledge on birth preparedness and complication readiness in rural setting Tanzania; A controlled quasi-experimental study
Source: PLoS One. 2021 Jan 12;16(1):e0244845. doi: 10.1371/journal.pone.0244845 (PMC7802973; doi:10.1371/journal.pone.0244845)
Supplement: Attachment — Submitted filename: Response to reviewers comments.docx [file pone.0244845.s005.docx]

**Point to point response to reviewers’ comments**

**Editor’s comment**

Include figure 2 in the text

**Response to the comment**

Figure 2 is cited in the text line 225

**Reviewer 1**

**Comment 1**

The author mentioned that the first household was selected using a random sampling approach, they selected the subsequent households using systematic sampling but no interval stated. this still needs clarity.

**Response 1**

Yes, the first household was picked randomly followed by systematic sampling. No interval was set but researchers move to already pre-determined direction. Each visited households were assessed for eligibility, if eligible was selected and next household was visited, if not eligible was skipped and next household was visited until the required sample size was reached

**Comment 2**

The authors have removed the sample size calculation but cited the published protocol, it would have been informative if a brief on this is given.

**Response 2**

Authors agree with the recommendation and the sample size calculation is added line 168-189

**Comment 3**

Verbal consent was done for women below 18 years, was there a justification why there was no assent? in some cases this group have been treated as mature minors and could provide their own consent. is there a justification why this is not the case in this population??

**Response 3**

Authors agree with the comment, it is true they are also taken as mature even in this population. What was done in the project were to seek consent from pregnant women then from their male partners; It was until both of them agree to participate then they were included in the study. The sentence is removed.

**Reviewer 2**

**Comment 1**

Thank you for addressing the comments and revising the manuscript. I also appreciate that the files have been added in supplemental information. Please note that during the proofing process, please conduct a thorough review, there are some errors that won't be caught by spell checker (i.e. line 240, Match for March, etc.)

**Response 1**

Line 240 is corrected
